# Through-drop imaging of moving contact lines and contact areas on opaque water-repellent surfaces†

**DOI:** 10.1039/d2sm01622b

**Published:** 2023-02-27

**Authors:** Arthur Vieira, Wenjuan Cui, Ville Jokinen, Robin H. A. Ras, Quan Zhou

**Affiliations:** a Department of Electrical Engineering and Automation, School of Electrical Engineering, Aalto University Maarintie 8 02150 Espoo Finland quan.zhou@aalto.fi; b School of Chemistry and Chemical Engineering, Lingnan Normal University Zhanjiang Guangdong 524048 P. R. China; c Department of Chemistry and Materials Science, School of Chemical Engineering, Aalto University Tietotie 3 02150 Espoo Finland; d Department of Applied Physics, School of Science, Aalto University P.O. Box 15100 02150 Espoo Finland robin.ras@aalto.fi; e Center of Excellence in Life-Inspired Hybrid Materials (LIBER), Aalto University P.O. Box 15100 02150 Espoo Finland

## Abstract

A myriad of natural surfaces such as plant leaves and insect wings can repel water and remain unwetted inspiring scientists and engineers to develop water-repellent surfaces for various applications. Those natural and artificial water-repellent surfaces are typically opaque, containing micro- and nano-roughness, and their wetting properties are determined by the details at the actual liquid–solid interface. However, a generally applicable way to directly observe moving contact lines on opaque water-repellent surfaces is missing. Here, we show that the advancing and receding contact lines and corresponding contact area on micro- and nano-rough water-repellent surfaces can be readily and reproducibly quantified using a transparent droplet probe. Combined with a conventional optical microscope, we quantify the progression of the apparent contact area and apparent contact line irregularity in different types of superhydrophobic silicon nanograss surfaces. Contact angles near 180° can be determined with an uncertainty as low as 0.2°, that a conventional contact angle goniometer cannot distinguish. We also identify the pinning/depinning sequences of a pillared model surface with excellent repeatability and quantify the progression of the apparent contact interface and contact angle of natural plant leaves with irregular surface topography.

## Abstract 2

The progressing wetting interface on opaque hydrophobic surfaces is observed and quantified using a transparent droplet probe and contact angles near 180° are measured with an uncertainty as low as 0.2°.

## Graphical abstract

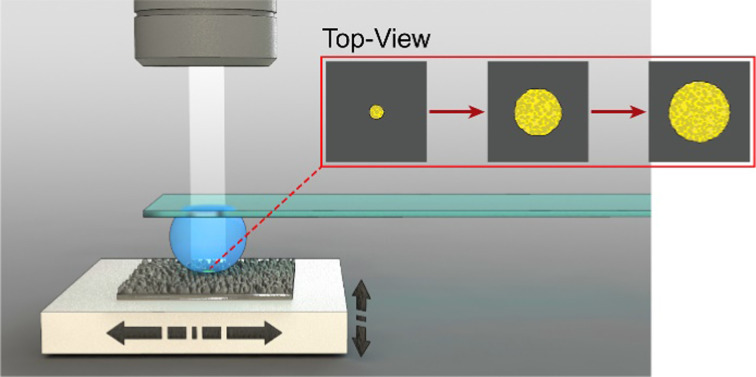

## 1. Introduction

Water-repellent, or hydrophobic, surfaces have attracted phenomenal attention due to the large number of examples found in nature, such as plant leaves^1^ and petals^2^ and insect legs and wings,^3^ and to their diverse applications in, for example, self-cleaning,^4^ oil–water separation,^5^ nano-assembly,^6^ anti-icing,^7^ and anti-foaming.^8^ Their water-repellent behaviour is often dictated by the chemical and micro- and nano-rough features of the surface at the wetting interface with the liquid.^9^ Observing the details of the wetting interface of droplets on surfaces, including the contact line (CL)^10,11^ and contact area morphologies,^12–15^ is key to understanding the wetting behaviour of water-repellent surfaces.^16^ Such understanding is crucial for developing advanced functional materials and devices, such as robust superhydrophobic surfaces^17,18^ and superhydrophobic 3D printed nanoporous objects.^19^

Most water-repellent surfaces are opaque and contain micro- and nano-rough features. Currently there is no effective method that can image and quantitatively analyse the advancing and receding wetting interface on those surfaces. Many methods for imaging the wetting interface can only partially image the CL and contact area or are limited to stabilized interfaces but not their progression. For example, an environmental scanning electron microscope (ESEM) provides high contrast imaging of the outer CL of droplets, provided the contact angle is low enough to avoid obscuring the CL,^10,20^ but provides no information of the internal contact area. Alternatively, the droplet and sample can be frozen under cryogenic scanning electron microscope (cryo-SEM)^14^ and destructively inspected using for instance a focused ion beam,^21^ to provide a static picture of the wetting state prior to freezing. In turn, X-ray tomography can provide detailed three-dimensional observation of the interface that reveals trapped air bubbles and heterogeneities at the droplet-sample interface, though imaging may take minutes.^15,22^ Force-based microscopy techniques, such as atomic force microscopy^23^ and scanning droplet adhesion microscopy,^24^ provide great probe controllability and can construct images of the wetting inhomogeneities of hydrophobic surfaces or the interfacial topography.^25^ However, force-based microscopy techniques do not reveal the progression of the interface. Optical imaging techniques are promising for measuring advancing and receding wetting interfaces. For example, confocal microscopy can observe changes to the CL and contact area on superhydrophobic samples^26,27^ and reflection interference microscopy can measure the thickness of the air layer trapped at the interface.^27,28^ These methods require the sample to be imaged through. However, most repellent samples are opaque and even when the materials themselves are transparent, the light scattering from the micro roughness makes imaging difficult. Immersion objectives^13^ and imaging through a sessile droplet^12^ can observe the interface regardless of sample transparency. Those methods, however, do not provide quantitative analysis of the CL progression because they either have poor image stability due to low droplet controllability or have a limited field-of-view. Contact angle goniometry (CAG) on the other hand, is the gold standard of surface wetting characterization, but cannot measure the CL and contact area. Moreover, despite its wide applications CAG suffers from high uncertainty due to optical and baseline positioning errors.^29^ The errors are most significant in the superhydrophobic regime, with uncertainties of multiple degrees for contact angles above 150°,^30^ limiting its applicability in this type of samples.

Here we report the direct imaging and quantitative analysis of the advancing and receding apparent CL on opaque micro- and nanorough surfaces using a transparent droplet probe. Using a normal optical microscope, our method reveals the details of the progression of the apparent contact area and apparent contact line irregularity at a video speed of 100 Hz. Advancing and receding contact angles between 160° and 180° are accurately calculated on superhydrophobic nanostructured surfaces, which CAG cannot distinguish due to an experimental standard deviation over 1°. By precisely controlling the volume and location of the droplet probe, experimental uncertainties as low as 0.2° are achieved. The contact angles were also measured using the transparent probe on a Digital Holographic Microscope (DHM), achieving similar uncertainty. Furthermore, the method was used to detect the pinning sequences on pillared surfaces with excellent repeatability and observe wetting interfaces on samples with a large variety of surface topographies, including natural samples with sophisticated wetting properties and artificial samples with defects.

## 2. Concept

**Fig. 1:**
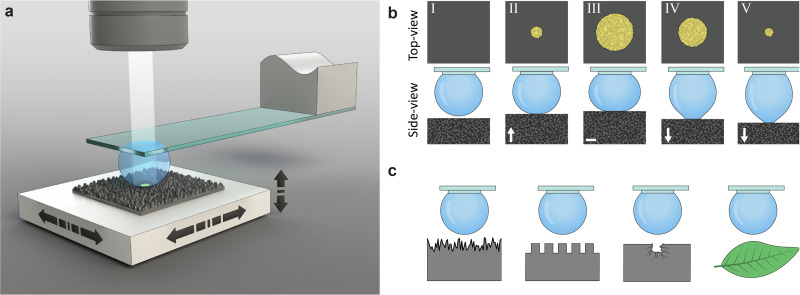
Transparent probe for through-droplet imaging of the wetting interface. (a) The transparent probe consisting of a liquid droplet pinned under a transparent glass slide can characterize water-repellent samples, such as a superhydrophobic silicon nanograss surface placed on an *XYZ* sample stage. During measurement, an optical microscope observes and records the evolution of the liquid–solid interface from above. (b) Measurement process. I – the droplet probe is placed above the sample but not in contact with it. II – the sample stage moves upwards, and the sample contacts the probe, resulting in an initial contact area. III – the sample stage moves further upwards and compresses the droplet, forcing the CL to advance. IV – after reaching a designated distance, the sample stage reverses direction. V – the sample stage moves further downwards, and the CL recedes until the droplet is detached from the sample surface. (c) The same procedure can measure a variety of surfaces, including silicon nanograss, micropillars, surface defects, and plant leaves. The illustrations are not to scale.

Our measurement method employs a water droplet probe fixed on a transparent holding disk underneath a glass slide (Fig. 1(a) and Fig. S2, ESI†). A sample is placed on a multi-axis precision-motorized sample stage that moves the sample in lateral and vertical directions, making the wetting experiments highly controllable and repeatable. For each measurement (Fig. 1(b)), the droplet is first filled to a set volume, and the sample stage moves laterally such that the droplet is above the measurement site of the sample. Then, the sample stage moves the sample upwards to the probe, creating an interface upon contact. The sample stage moves further upwards, increasing the contact area, and then reverses. The downwards motion causes the interface to shrink, and eventually the sample is detached from the droplet, ending the measurement. The probe is fixed under an optical microscope that images the liquid–solid interface through the probe during both the growing and shrinking of the interface. The recorded images provide quantitative information, such as the location of the advancing and receding apparent contact area and CL and pinning and depinning events. The same transparent probe can image the wetting interface (*i.e.*, contact line and contact area) on samples with rough surfaces, including silicon nanograss, micropillars, defective surfaces, and biological samples such as plant leaves (Fig. 1(c)).

## 3. Results and discussion

### 3.1. Advancing and receding contact area, contact line irregularity and contact angle

**Fig. 2:**
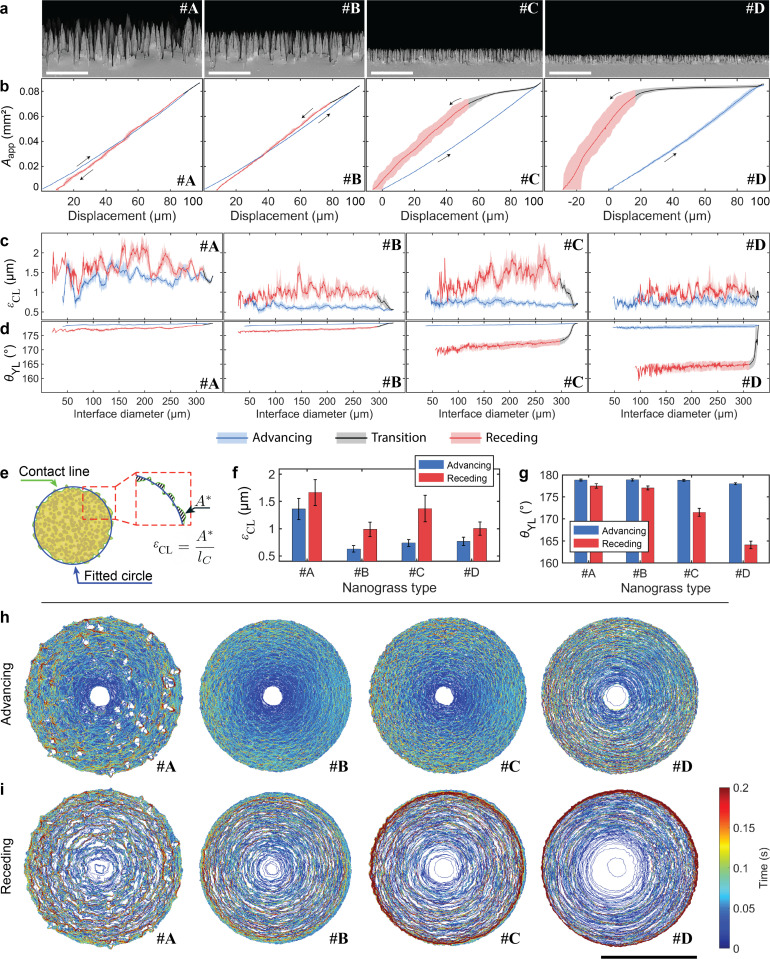
Advancing and receding contact lines and contact areas on four different types of silicon nanograss surfaces, labelled #A–#D. Measurements were obtained at the same location on the respective samples and repeated 10 times. (a) SEM images of different types of silicon nanograss. (Scale bars: 5 μm) (b) Apparent contact area *A*_app_ as a function of sample stage displacement for each type of silicon nanograss; the solid lines represent the mean, and the shaded area is bounded by the minimum and maximum values of observations. (c) Apparent contact line irregularity *ε*_CL_ and (d) contact angle *θ*_YL_ obtained from Young–Laplace calculation, as a function of interface diameter. (e) Illustration of *ε*_CL_ calculation based on the top-view image. A circle (blue line) was fitted to the CL (green line); the inner and outer differences in area A* were calculated, shown as the dashed area in the inset, and then divided by the perimeter of the fitted circle, *l*_C_. (f) Mean and standard deviation of *ε*_CL_ during the advancing and receding phases. (g) Mean and standard deviation of advancing and receding *θ*_YL_, for each type of nanograss. (h) Advancing and (i) receding CL progression for one measurement. The color indicates how long the CL was present at each pixel. (Scale bar: 200 μm).

We measured the advancing and receding wetting interface on four types of superhydrophobic silicon nanograss, labelled #A, #B, #C and #D. The four types of silicon nanograss differed in height from approximately 5.0 μm (nanograss #A) to 1.1 μm (nanograss #D), as shown in Fig. 2(a) and Fig. S3, S4 (ESI†). They also differed from each other in the density of spikes (Table S1, ESI†). However, the advancing and receding contact angles for all four types were approximately 170°, when measured with a commercial contact angle goniometer (Table S2, ESI†), where the difference between types was smaller than the uncertainties of the measurements.^30,31^ The measurements were performed at a single location repeated 10 times (Fig. 2, Fig. S5 and Movie S1, ESI†) and also at 10 different locations (Fig. S6, ESI†).

To quantitatively analyse the interface progression, we developed a machine vision algorithm to detect the contact area and perimeter (see Materials and methods and algorithm S1, ESI†). The apparent contact area, *A*_app_, was measured as a function of sample stage displacement, shown in Fig. 2(b). We split the measurement results into advancing, transition, and receding phases. During the advancing phase, *A*_app_ grew smoothly and highly repeatably for all samples in both the single location and different locations measurements.

After the sample stage reverses direction, *A*_app_ did not necessarily shrink proportionally to displacement. We define the transition phase during which the CL resisted movement (see Fig. S9, ESI† for details), present in all nanograss types (black section of the curves in Fig. 2(b)–(d)). In particular, nanograss #D showed little change during this phase, suggesting CL pinning.

The receding phase follows the transition phase. For all samples, *A*_app_ exhibited greater variations during the receding phase compared to the advancing phase (Fig. 2(b) and Fig. S5a, ESI†). The greater shaded area in nanograss in #C and #D, in Fig. 2(b), is not the result of random variation but of a trend in the displacement area relation (Fig. S5a, ESI†). We attribute this to the degradation of the sample surface after being repeatedly touched by the droplet, since the variations are related to the number of measurements. Nevertheless, the curves are similar, demonstrating that the droplet was affected by the same chemical and topographical inhomogeneities.

We also determined the apparent contact line irregularity, *ε*_CL_, which describes the arithmetic average deviation of the CL from a circle. The calculation of *ε*_CL_ is illustrated in Fig. 2(e) and discussed in detail in Section 2 (ESI†). We measured *ε*_CL_ during advancing and receding of the CL (Fig. 2(c)). The measurement shows that nanograss #A experienced more chaotic CL progression, attributed to the greater gaps between spikes. Despite the chaotic CL evolution, it is noticeable that *A*_app_ is rather smooth and repeatable, and the transition phase is small (see nanograss #A in Fig. 2(b)). We attribute this to the lower density of interface features touching the droplet, leading to sparser depinning events (Fig. 2(a) and Table S1, ESI†). Nanograss #D showed an approximately constant *ε*_CL_ during the transition phase, suggesting that the CL was fully pinned (see also nanograss #D in Fig. S5c, ESI†). The mean and standard deviation of *ε*_CL_ during advancing and receding is shown in Fig. 2(f) for each type of nanograss. Except for nanograss #A, we found the advancing *ε*_CL_ to be significantly lower than the receding *ε*_CL_. The higher *ε*_CL_ values result from the chaotic movement of the CL as it depins from each surface feature.

The repeatability and precision of the method allows us to numerically calculate the apparent mean contact angle, *θ*_YL_, during the whole measurement, shown in Fig. 2(d). The simulations use axisymmetric Young–Laplace equation to solve the shape of the droplet from which the contact angle is derived (see Section 1 and Fig. S1 for details, ESI†). We calculated the mean and standard deviation of the advancing and receding phases of *θ*_YL_, shown in Fig. 2(g). The advancing *θ*_YL_ is 178.9 ± 0.2°, 179.0 ± 0.2°, 178.8 ± 0.2° and 178.0 ± 0.3°, for nanograsses #A, #B, #C and #D respectively, (Table S2, ESI†). We attribute this to a similar mechanism to that studied by Schellenberger *et al.* on pillared surfaces,^26^ where they demonstrate that the CL advances as the liquid–air surface gradually bends down, leading to a macroscopic contact angle near 180°. On the other hand, the receding *θ*_YL_ varies across the different types of nanograss, with progressively smaller mean value: 177.7 ± 0.5°, 177.3 ± 0.4°, 171.9 ± 0.7° and 164.3 ± 0.4° for nanograss #A, #B, #C and #D respectively. We note that the main source of uncertainty in the calculation of *θ*_YL_ for each frame comes from the error in the measurement of the interface radius in our top-view machine vision analysis. In our method the sensitivity of *θ*_YL_ to an error of one pixel in the estimation of the interface radius is ∼0.1° per px near *θ*_YL_ = 180° (see Fig. S15, ESI†). In comparison, commercial CAG can have an error of multiple degrees per each pixel of error in the positioning of the baseline, which defines de plane where the droplet touches the sample.^29^

Complementary to the contact angle values produced by the Young–Laplace model using a conventional optical microscope, we also used the transparent probe on a DHM to directly measure the contact angle from the 3D profile of the droplet and wetting interface, available in Table S2 (ESI†) (see Materials and methods and Fig. S7, ESI† for Experimental details). The results show that the advancing contact angle on all samples is indeed >178° and the receding contact angles were 178.8 ± 0.5° and 178.6 ± 0.4° for nanograss #A and #B respectively, confirming their superhydrophobicity and low contact angle hysteresis. The receding contact angles on nanograss #C and #D were not possible to measure due to technical limitations of the DHM related to the maximum measurable sample slope angle.

We also produced CL progression maps of the movement of the CL during advancing and receding, shown in Fig. 2(h) and (i) respectively. These maps reveal how the CLs move differently on each sample despite having similar topographical features. The advancing CL in nanograss #A shows many interaction sites where we interpret the CL wraps around the highest spikes of the sample (see also Video S1 and Fig. S8, ESI†). Surprisingly, nanograss #B shows the most continuous advancing CL, which agrees with the lowest mean advancing ε_CL_ in Fig. 2(f). Nanograss #B has very similar wetting properties to #A, with similarly high contact angle and low contact angle hysteresis. However, its features are just below the camera's pixel size, of approximately 0.75 μm (Table S1, ESI†) and individual interaction sites cannot be resolved. The CL in nanograss #C advances similarly to nanograss #B but more irregularly, while in #D it advances in concentric steps. During receding the CL moves in irregular stepwise manner for all nanograss types, due to pinning on surface features (Fig. 2(i)). A trend is observed where the time spent by the CL at the maximum perimeter *versus* the interior of the interface increases from nanograss #A to #D. We also note that the CL progressions in Fig. 2(i) include both the transition and receding phases. For this reason, nanograss #C and #D show signs of the CL pinning at the perimeter of the interface during the transition phase. At the same time, the CL in nanograsses #C and #D spends less time in the center regions, with #D detaching from the droplet in only a few frames.

Additionally, we imaged the evolution of the wetting interface when the droplet probe was sliding on a silicon nanograss surface containing a scratch (Fig. S13, ESI†). We were able to observe how the scratch affected wetting when it entered and exited the interfacial region, which provides useful information for studying the influence of defects on wetting. A video recording of the experiments can be found in Movie S4 (ESI†).

### 3.2. Pinning and depinning sequence on pillared surfaces

**Fig. 3:**
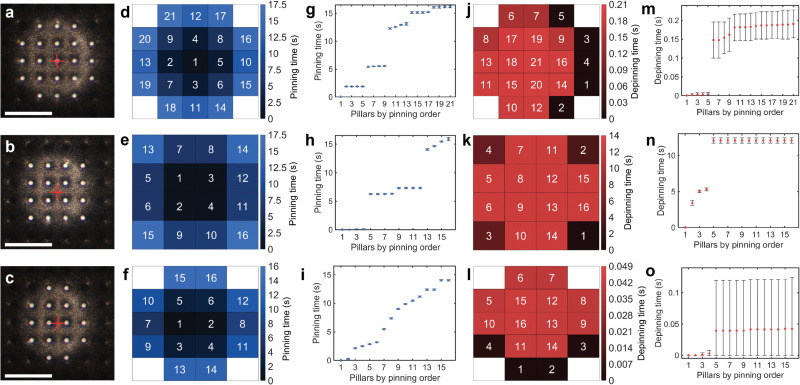
Pinning and depinning order on silicon micropillars. Measurements were performed for three droplet-pillar alignment cases and repeated 10 times. (a)–(c) Top-view of the pinning experiments; where the contacting pillars are bright, the red cross denotes the initial position of the center of the droplet. (Scale bar 200 μm) (d)–(f) Maps of the pinning order of each pillar. The numbers in the maps indicate the pinning order, and the colors represent the pinning time. (g)–(i) Mean pinning times and standard deviation relative to the first pinning pillar. (j)–(l) Maps of depinning order. The numbers in the maps indicate the depinning order, and the colors represent the depinning time. (m)–(o) Mean depinning times and standard deviation relative to the first depinning pillar; the initial alignment of the probe was centered on one pillar (a), (d), (g), (j), (m), the middle of four pillars (b), (e), (h), (k), (n), and the middle of two pillars (c), (f), (i), (l), (o).

To evaluate the sensitivity and repeatability of our method, we observed the pinning and depinning events on a water-repellent micropillar model surface with a pillar diameter of 20 μm, period of 80 μm and height of 44 μm (Fig. 3, Fig. S10 and Table S4, ESI†). We varied the initial alignment between the droplet and the pillars in three cases, centered on one pillar, Fig. 3(a), the middle of four pillars, Fig. 3(b), and the middle of two pillars, Fig. 3(c) (see also Movie S2, ESI†).

The experimental results demonstrate excellent time synchronization of pinning events in many cases. For example, in the case where the droplet was centered on one pillar, after first touching the center pillar, the four neighbouring pillars (order number 2–5) always pinned within 10 ms on all 10 measurements, Fig. 3(d) and (g). The difference in droplet-pillars alignment leads to dramatic differences in the sequence and number of pinning events; for example, in the case where the droplet was aligned between four pillars, Fig. 3(b), only 16 pillars were contacted for the same sample stage displacement, as opposed to 21 pillars in the centered case, Fig. 3(a). In the mid-four case, synchronization in groups was also similar, except for the last group, which took approximately 1.82 s for all four pillars to pin (order number 13–16 in Fig. 3(h)). For the mid two case of Fig. 3(c), there was little synchronization between pinning events due to initial misalignment of the droplet from the mid-point between two pillars. In this case, there was no simultaneous group of pillars at the same distance to the geometric center of the droplet. However, the individual pinning events of each pillar remain highly repeatable, demonstrating that our method can detect the influence of minor pillar misalignment.

Depinning events are mostly an abrupt process compared to pinning, with all pillars depinning almost simultaneously (Fig. 3(m)–(o)). The initial alignment of the droplet with the sample also dramatically affects the depinning sequence and duration. In the centered and mid two cases, depinning was almost simultaneous, with all depinning occurring in about 50 ms and 200 ms, respectively. We note that in mid two case, Fig. 3(o), the top error bars represent the standard deviation of the measurements, while the bottom error bars are limited at zero. In the mid-four case, it took over 12 s for all pillars to depin following the depinning of the first outer pillar. Nevertheless, depinning also exhibits extremely good repeatability, with all events occurring within 370 ms.

### 3.3. Imaging of wetting interface on biological leaves and scratched surface

**Fig. 4:**
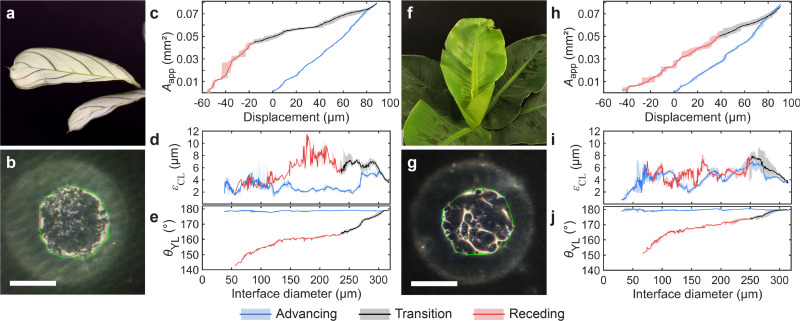
Imaging of wetting interface on plant leaves. All measurements were obtained at the same location of the respective samples and repeated 10 times. (Scale bars: 200 μm) (a) Image of a Maranta leaf. (b) Optical micrograph of the wetting interface between the droplet probe and the Maranta leaf; the green line encloses the detected apparent contact area. (c) Evolution of apparent contact area *A*_app_ as a function of sample stage displacement for the Maranta leaf; the solid line represents the mean, and the shaded area is bounded by the minimum and maximum values of observations. (d) Progression of apparent CL irregularity *ε*_CL_ and (e) Young–Laplace contact angle, *θ*_YL_, as a function of interface diameter. (f) Image of a Musa (Oriental Dwarf) leaf, and the respective (g) wetting interface, (h) apparent contact area, (i) apparent contact line irregularity and (j) Young–Laplace contact angle.

We also explored the applicability of the transparent droplet probe to biological samples by measuring the progression of the wetting interface on two types of plant leaves, Maranta and Musa (see Fig. 4 and Fig. S11, ESI†). The Maranta's surface is flatter overall, with smaller scale features, while the Musa exhibits greater surface roughness and topographical variations; see Fig. S12 and Table S5 (ESI†).

Example snapshots of the evolution of the wetting interfaces are shown in Fig. 4(b) and (g), where the apparent contact area algorithmically identified is enclosed by a green line (see Movie S3, ESI† for more details). As Fig. 4(b) and (g) show, the apparent wetting interfaces of both leaves are rather inhomogeneous. The interface of the Maranta consists of many subtle variations, while the interface of the Musa is dominated by fewer but more significant variations, which we attribute to surface topography features.

The progression of *A*_app_ as a function of sample stage displacement is shown in Fig. 4(c) for the Maranta leave and Fig. 4(h) for the Musa leave (see also Fig. S11, ESI†). Despite the surface inhomogeneities of the leaves, it is surprising to see that *A*_app_ is rather reproducible in both cases. Both leaves demonstrate high but reproducible *ε*_CL_ value, Fig. 4(d) and (i) for the Maranta and Musa respectively. Maranta leaf has a mean *ε*_CL_ of 3 μm/6 μm (advancing/receding) and for Musa a *ε*_CL_ of 5 μm/5 μm (advancing/receding), which we attribute to their microscale topographical features (see Table S6, ESI†). We also calculated the *θ*_YL_ for both plant leaves, shown in Fig. 4(e) and (j). The mean advancing *θ*_YL_ were 178.6 ± 0.3° and 179.7 ± 0.4° and the mean receding *θ*_YL_ were 159 ± 4° and 168 ± 5°, for Maranta and Musa leaves respectively. The resulting contact angle hysteresis were 20 ± 5° and 12 ± 5° respectively, indicating that the Musa leaf is more hydrophobic than the Maranta leaf.

The advancing contact angle values measured with our method are more than 10° greater than those obtained using CAG: advancing 166.9 ± 3.7° and 166.5 ± 4.0° and receding 166.4 ± 4.2° and 167.7 ± 1.7° for Maranta and Musa leaves respectively. The lower advancing CAG contact angle values can be attributed to the non-flatness of the leaves (see Fig. S12, ESI†), which obscures the actual CL. The non-flatness can also contribute to the greater CAG uncertainty due to the dependence of the baseline on the CL position. On the other hand, the non-flatness and inhomogeneous wetting properties of the plant leaves lead to greater uncertainty also in our *θ*_YL_ model, which reflects mostly in the receding contact angle values.

## 4. Conclusions

By imaging through the droplet, we developed a method that allows facile direct observation of the progression of the wetting interface with excellent detail and repeatability. The method is flexible and can be applied to a variety of micro- and nano-rough water repellent samples, both artificial and natural, without requiring the sample to be transparent. Moreover, we show the method can be applied on two different types of microscopes, a regular optical microscope as well as DHM. The through-drop imaging method can provide rich information on the progression of the interface, including time series data on the apparent contact area *A*_app_ and contact line irregularity *ε*_CL_. The time resolution is only limited by the acquisition speed of the camera, in our case 100 Hz. Considering many wetting events happen in milli-seconds, a high acquisition rate is important. The repeatability and precision of through-drop imaging allow the measurement of the apparent mean contact angle *θ*_YL_ with a significantly smaller error than contact angle goniometry. This allows distinguishing samples with similarly high contact angles that would otherwise be reported as the same.

The great sensitivity and repeatability of our method may open new doors for the scientific study of water-repellent surfaces. Considering its capabilities and relatively easy implementation, our method may be easily adopted. We expect our method to benefit scientists and engineers studying repellent surfaces in multiple disciplines, including biology, material science, interfacial science, pharmaceuticals, civil engineering, and manufacturing.

## 5. Methods and materials

### 5.1. Measurement procedure

Prior to each measurement, a water droplet was dispensed onto the probe with a volume above the target 1.5 μL. The volume was estimated using a custom image analysis algorithm from the side-view images at the start and end of each measurement, to account for the effects of evaporation. The droplet was then discharged by touching an electrically grounded piece of nanograss #A with an approximately 110 μm scratch to facilitate electrical contact. Following that, the droplet was moved above the measurement site by laterally moving the sample. The measurement then started by moving the sample surface towards the droplet by raising the sample stage at a constant speed, pressing the droplet up to 100 μm. Retraction of the sample was performed by lowering the stage at the same speed until the droplet separated from the sample. The silicon nanograss and plant leaf measurements were obtained with a velocity of 10 μm s^−1^, and the pillar measurements were obtained with a velocity of 5 μm s^−1^. These velocities were selected to be as low as possible to observe rich features and details, while keeping the effects of evaporation negligible during a measurement (see Fig. S14, ESI† for details). All measurements were taken under room conditions: a temperature of 24–25 °C and 13–40% relative humidity.

### 5.2. Apparatus

The top view and side view images were obtained by cameras operating at 100 frames-per-second and at a resolution of 1464 × 1464 px^2^ using a variable zoom lens (VZM 600i, Edmund Optics Inc., USA) with 1–6× magnification; two models of cameras were used: a monochromatic BFS-U3-28S5M C and a color BFS-U3-28S5C C, Flir LLC. For the silicon nanograss experiments, both the top-view and side-view were monochromatic; for the pillar experiments, both the top-view and side-view were color, and for the plant leaves experiments, the top-view camera was color, and the side-view camera was monochromatic. For coaxial illumination, a 15 mm 50R/50T standard cube beam splitter (Edmund Optics Inc., USA) was assembled on the top-view lens with a custom mount. The probe was mounted beneath the top-view camera, with a 5.5° tilt angle to prevent direct reflection (see inset of Fig. S2, ESI†). The light source used was model OSL2, with a collimating lens (OSL2COL, Thorlabs Inc., USA). Each sample was mounted on a three-axis precision motorized stage, models M-404.8PD, M-122.2DD and M-111.1DG, for the *x*, *y*, and *z* axes, respectively, from Physik Instrumente GmbH, Germany. The top-view camera was mounted on a precision motorized stage (model M-122.2DD, Physik Instrumente GmbH, Germany) for focus tracking. To measure the *z* axis sample displacement, a laser interferometer, (model IDS3010) with a fibreoptic sensor head (model D4/F17, Attocube Systems AG, Germany) was installed above the sample stage. A reflective silicon wafer cut-out was placed on the sample stage, providing a reflective surface for the interferometer. The liquid droplet probe was formed with a nanoliter dispenser PipeJet from BioFluidix GmbH, Germany to dispense water. Type 1 ultrapure water was used in all measurements, with a resistivity of 18.2 MΩ cm, obtained with Direct-Q® 3 UV Water Purification System, Milli-Q. Several custom 3D-printed parts were produced using a stereo-lithography printer (model SL1, Prusa Research a.s., Czech Republic) from liquid resin Strong-X, by Liqcreate, Netherlands. To collect the data, a data acquisition board was used, (model NI USB 6363, National Instruments Inc., USA). The setup was controlled by custom software.

### 5.3. Transparent probe

A #0 glass coverslip was cut into 22 × 3 mm slices using a Disco DAD3220 dicing machine. A small droplet (approx. 30 nL) of Delo GB310 UV curing glue was placed at one end of the glass slide with a remote-controlled robotic manipulator using the end of a 0.40 mm copper wire to contact dip the glue. A 1 mm diameter SU-8 undercut disk^32^ was placed over the glue droplet with the help of a custom robotic gripper. The glue was cured with a UV curing light source (model Blue Wave 50, Dymax Europe GmbH). To fabricate the SU-8 undercut disks, a 100 μm thick layer of SU-8 50 was spin-coated (1500 rpm, 30 s) on a silicon oxide coated wafer followed by 15 min at 95 °C soft bake on a hotplate, 20 s UV exposure using a Karl Suss MA6 mask aligner, 15 min post exposure bake at 95 °C and then development in PGMEA for 20 min. The disks were manually released using a scalpel.

### 5.4. Silicon nanograss coated with fluoropolymer

The silicon nanograss was produced by a maskless cryogenic deep reactive ion etching process using an Oxford Plasmalab System 100 on a 4 inch silicon wafer (〈100〉, p-type boron doped, >1 Ω cm). The process parameters were 1000 W of ICP forward power, a temperature of −110 °C, 10 mTorr of pressure, and 7 minutes of etching time. Varying etching gas flow rates and forward powers were utilized to obtain the different nanograss morphologies. For nanograss #A, #B, #C, and #D, the SF_6_ gas flow rate was 40.0, 37.6, 35.3 and 32.9 sccm, respectively; the O_2_ gas flow rate was 18.0, 20.4, 22.8, and 25.1 sccm, respectively, and the forward power was 6, 6, 5, and 4 W, respectively. All etched silicon nanograss samples were coated with a thin fluoropolymer film for superhydrophobicity by plasma-enhanced chemical vapor deposition (PECVD) using an Oxford Plasmalab 80+ with 100 sccm of CHF_3_ for 5 minutes under 250 mTorr of pressure and 50 W of forward power.

### 5.5. Silicon micropillars

The silicon micropillars were fabricated by cryogenic deep reactive ion etching with a silicon dioxide hard mask. The starting substrate was a 〈100〉 silicon wafer with 500 nm wet thermal oxide. The micropillar pattern was defined by UV lithography (AZ5214 photoresist, Süss MA6 mask aligner). The oxide was etched with reactive ion etching using an Oxford Plasmalab 80+ (Oxford Instruments, Bristol, UK), 18 min etching time, 200 mTorr pressure, 30 W power, 25 sccm CHF_3_, and 25 sccm Ar flows. The photoresist was then stripped by ultrasonication in acetone for 10 min. Next, the micropillars were etched using an Oxford Plasmalab 100 (Oxford Instruments, Bristol, UK). The micropillars were fabricated with an anisotropic silicon etch (O_2_ 6 sccm, SF_6_ 40 sccm, forward power 3 W, ICP power 1050 W, 110 °C temperature, 8 mTorr pressure, 24 min etching time). Afterwards, the oxide mask was stripped in buffered HF. Finally, the pillars were coated with a thin fluoropolymer film for superhydrophobicity by plasma-enhanced chemical vapor deposition (PECVD) using an Oxford Plasmalab 80+ with 100 sccm of CHF_3_ for 5 minutes under 250 mTorr of pressure and 50 W of forward power. The etch depth was determined by SEM image to be approximately 44 μm. The diameter of the pillars was 20 μm, and the pillars were in a square array with period 80 μm.

### 5.6. Plant leaves

Maranta (*Leuconeura Amabilis Mint*) and Musa ‘Oriental’ Dwarf (*Musa acuminata*) potted plants were bought fresh from local sellers. Test specimens were cut using scissors and attached to a glass slide using double-sided tape. Prior to each measurement the samples were rinsed using purified water. The confocal measurements were obtained using an S neox 3D optical profilometer (SensoFar Metrology) in confocal mode with a 20× objective.

### 5.7. SEM imaging

For scanning electron microscope imaging, silicon nanograss samples were cut and coated with 5 nm of gold–palladium using a Leica EM ACE600 high vacuum sputter coater. Images were taken with a Zeiss Sigma VP SEM, with an acceleration voltage of 2 kV, in high vacuum mode using an in-lens secondary electron detector. The etch depth and spike tip radii were measured manually from side-view images using Adobe Illustrator.

### 5.8. AFM imaging

AFM images of silicon nanograss samples were obtained using a Dimension Icon AFM (Bruker Corporation) in ScanAsyst mode with a ScanAsyst-Air probe. The spike density and spacing were determined from AFM images using Gwyddion software.

### 5.9. Image analysis of apparent contact area and contact line

To estimate the interfacial area and apparent contact line irregularity, the top-view camera recordings were analysed using a custom MATLAB script. In all measurements, the first frame was analysed independently using binary threshold with a fixed value, which identifies the center-most bright spot reflecting from the bottom of the droplet prior to contact. Subsequent frames were then analysed iteratively. For each new frame, a search-area was defined as the morphological dilation^33^ of the convex hull of the apparent interface area found in the previous frame. Within this search-area, the wetting interface was also identified with binary threshold. The morphological operator used in dilation and the threshold value applied within the search-area are chosen differently depending on the sample.

For the silicon nanograss samples, the same threshold value was used in all frames. The value was chosen using Otsu's method^34^ based on the values of the pixels in the frame where displacement of the sample stage was the highest, *i.e.*, the droplet was pressed the most and thus the interface area was the largest. The morphological operator used to define the search area was a disk with a radius of 30 pixels. The largest blob was selected and filled. For the plant leaves, a different threshold value was used on each frame, using Otsu's method based on the contents of the search area. Additionally, a different morphological operator was used during the pressing and depressing of the droplet. For the Maranta leaf, the pressing morphological operator was a disk with a 10 pixel radius, and no dilation was performed on depressing. For the Musa leaf, the pressing morphological operator was an asymmetric ellipsoid, and the depressing morphological operator was a disk with a radius of 25 pixels. The algorithm is summarized in Algorithm S1 (ESI†). The interface features detected within the search area were connected using a modified snakes algorithm.^35^

For all samples, the apparent contact line irregularity was estimated by fitting a circle to the perimeter of the detected area. Deviations between the perimeter and the fitted circle were integrated around the circle and normalized to the perimeter of the fitted circle.

### 5.10. Micropillar image analysis

To identify the pinning and depinning events, we analysed the top-view camera recordings with a custom MATLAB script on 10 measurements for each alignment case: centered on one pillar, between two pillars, and between four pillars. First, a frame where all pillars were in contact with the droplet was used to generate a binary mask, later used in other frames to isolate the pillars of interest from the rest of the image. An iterative analysis method was used to identify pinning/depinning pillars. If a sufficiently large difference between consecutive frames was detected inside each pillar's masked region, an event was identified. Pinning maps were built using the absolute difference between frames during the upwards motion of the sample stage. To build a depinning map, only decreases in brightness in the masked pillars during the downwards motion of the sample stage were considered.

### 5.11. Contact angle goniometry

The contact angle measurements were obtained with an Attension® Theta Lite optical tensiometer (Biolin Scientific) with an automatic dispensing system. A droplet of approximately 2 μL was dispensed and placed in contact with the sample. For measuring the advancing contact angle, the volume was increased at a rate of 0.05 μL s^−1^ for 70 s. For measuring the receding contact angle, the volume was decreased at a rate of 0.05 μL s^−1^ for 70 s. All measurements were repeated 10 times.

### 5.12. Digital holographic microscopy

The contact angle measurements of nanograss were verified using R-1000 reflection DHM with an objective with ×10, NA 2.8, Lyncée Tec SA. The probe was mounted parallel to the sample. A 1.5 μL droplet was dispensed on the transparent probe. The droplet was moved into contact with the sample at a speed of 10 μm s^−1^ and retracted at the same speed. Two 3D frames were chosen for analysis, one during the advancing of the CL and one during receding, which were analysed using a MATLAB script. The advancing and receding contact angles were measured around the CL and averaged.

## Author contributions

Q. Z. and A. V. conceived the research. Q. Z., A. V. and R. H. A. R. designed the method. Q. Z., A. V., W. C. designed the experiments. A. V. developed the apparatus and algorithms and performed most of the experiments and quantitative analysis. All authors analysed the data. W. C. prepared the silicon nanograss samples. V. J. prepared the silicon pillared samples. W. C. helped with the contact angles and the SEM and AFM measurements of the samples. Q. Z. and R. H. A. R. supervised the research. All authors discussed and co-wrote the paper.

## Conflicts of interest

There are no conflicts to declare.

## Supplementary Material

SM-019-D2SM01622B-s001

SM-019-D2SM01622B-s002

SM-019-D2SM01622B-s003

SM-019-D2SM01622B-s004

SM-019-D2SM01622B-s005
