# Supplementary material for: Through-drop imaging of moving contact lines and contact areas on opaque water-repellent surfaces
Source: Soft Matter. 2023 Feb 27;19(13):2350–9. doi: 10.1039/d2sm01622b (PMC10053025; doi:10.1039/d2sm01622b)
Supplement: SM-019-D2SM01622B-s005 [file SM-019-D2SM01622B-s005.pdf]

Electronic Supplementary Material (ESI) for Soft Matter.
This journal is © The Royal Society of Chemistry 2023

Supplementary Information

Through-drop imaging of moving contact lines and contact areas on opaque water-repellent surfaces

Arthur Vieira,[a] Wenjuan Cui,[a,b] Ville Jokinen,[c] Robin H. A. Ras*[d,e] and Quan Zhou*[a]

[a] *Department of Electrical Engineering and Automation, School of Electrical Engineering, Aalto University, Maarintie 8, 02150 Espoo, Finland*

[b] *School of Chemistry and Chemical Engineering, Lingnan Normal University, Zhanjiang, Guangdong 524048, P.R. China*

[c] *Department of Chemistry and Materials Science, School of Chemical Engineering, Aalto University, Tietotie 3, 02150 Espoo, Finland*

[d] *Department of Applied Physics, School of Science, Aalto University, P.O. Box 15100, 02150 Espoo, Finland*

[e] *Center of Excellence in Life-Inspired Hybrid Materials (LIBER), Aalto University, P.O. Box 15100, 02150 Espoo, Finland*

* Corresponding authors: quan.zhou@aalto.fi, robin.ras@aalto.fi

1. Computational model for contact angle

To compute the contact angle for each frame of the measurement we assume an axisymmetric droplet profile shown in Fig. S1. We also assume that the droplet shape is always in equilibrium and gravity can be neglected. Under these conditions the droplet's profile is given by Young-Laplace equation:

$$\frac{u''}{\left(1+u'^2\right)^{3/2}} - \frac{1}{u\sqrt{1+u'^2}} = -\Delta P/\gamma \tag{1}$$

where $u = u(z)$; u' and u'' are the first and second derivative of $u(z)$; ΔP is the Laplace pressure and γ the surface tension of water.

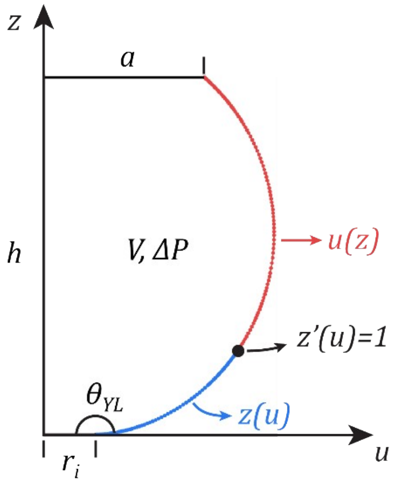

Fig. S1 Schematic of the axisymmetric Young-Laplace problem. The profile of the water-air interface is defined by $u(z)$. a – radius of the droplet-holding-disk. r_i – radius of the interface with the sample. h – distance between sample and droplet-holding-disk. V- droplet volume. ΔP- Laplace pressure. A double branch integration is used. First $z(u)$ is integrated from $z = 0$ until $z'(u) = 1$, blue line, and from that point on $u(z)$ is integrated until $z = h$, red line.

The interface radius, r_i, radius of the disk, a, and droplet height, h, form a boundary value problem:

$$\begin{cases} \dfrac{u''}{\left(1+u'^2\right)^{3/2}} - \dfrac{1}{u\sqrt{1+u'^2}} = -\Delta P/\gamma \\ u(0) = r_i \\ u(h) = a \\ \displaystyle\int_0^h \pi u^2 dz = V \end{cases} \tag{2}$$

where the contact angle can be obtained as $\theta_{YL} = tan^{-1}(u'(0))$.

To solve for $u(z)$ we frame the problem as an initial value problem defined by $u(0)$ and $u'(0)$. We use a double shooting method to first find θ_{YL} that solves $u(h) = a$ and then find ΔP that solves

for the correct volume V. Due to errors in measuring r_i, h and V, θ_{YL} can in some cases exceed 180° for which $u(z)$ would no longer be a defined function. To account for this we integrate $u(z)$ in two branches, shown in blue and red in Fig. S1. The first branch is integrated using the inverse equation for $z(u)$ (Eq. 3), from $z = 0$ until $z^{'}(u) = 1$, and from there on using $u(z)$ (Eq. 2) until $z = h$.

$$\frac{z''}{\left(1 + z^{'2}\right)^{3/2}} - \frac{z^{'}}{u\sqrt{1 + z^{'2}}} = -\Delta P/\gamma \tag{3}$$

with $z = z(u)$; $z^{'}$ and $z^{''}$ the first and second derivatives of $z(u)$.

The contact radius is measured from the top-view camera, by fitting a circle to the contact interface, which is identified through the machine vision algorithm, described in Algorithm S1. The disk radius was measured under the microscope to be $a \simeq 511\ \mu m$. The volume is controlled during the experiment to be approximately $1.5\ \mu L$. In post-processing, the volume is measured from side-view camera at the start and end of the experiment, to account for evaporation. The volume during the experiment is estimated by linear interpolation. We set the initial height to be that of an ideal spherical droplet attached to the disk, based on the initial volume at the moment of first contact. The sample-to-disk height h is continuously measured with a laser-interferometer displacement sensor (Fig. S2), relative to the initial height.

2. Contact Line Irregularity

To calculate the contact line irregularity, ε_{CL}, first the outline of the wetting interface is obtained through machine vision and a circle is fitted to it (see Fig. 2e). The deviations from the circle are integrated around the perimeter, A^*, and normalized to the perimeter of the fitted circle l_C:

$$\varepsilon_{CL} = \frac{A^*}{l_C} \#(4)$$

This is analogous to the calculation of topographical roughness R_a[1].

$$R_a = \frac{1}{L}\int_0^L |z(x)|dx \#(5)$$

where $z(x)$ is the surface height profile, measured from the mean line and L is the length along which the integral is taken. Where as R_a is a measure of topographical height along in the z axis, ε_{CL} can be interpreted as the CL roughness along the wetting interface perimeter, in the plane of the interface xy.

Algorithm S1 - Pseudo-code for wetting interface identification

Inputs: Video file V containing frames f_i; i_{peak} index of highest sample stage position; $m1$ and $m2$, morphological operators.

Output: `a[]`, interface outline for each frame.

```
a = {}
a[0] ← T_0.5(f_0)
for each f_i ∈ V : i ≥ 1
    if i < i_peak
            s = a[i-1] ⊕ m1 // Sample stage approaching
    else
            s = a[i-1] ⊕ m2 // Sample stage retracting
    end
    b = T_otsu(f_i, s)
    if sample is plant leaf
            a[i] ← Snakes(b)
    else if sample is nanograss
            a[i] ← boundary(b) // Boundary of biggest blob
    end
end
```

Pseudo-code for estimating droplet-sample contact interface from top-view video frames. $T_{0.5}(f_0)$ is the binary threshold with a fixed value 0.5, in range of 0 to 1, applied to the first frame f_0; $T_{otsu}(fi, s)$ is binary threshold following Otsu's method[2], applied within search area s in frame fi; $Snakes(b)$ is a modified snakes active contour function[3] applied to the blobs in b; $\oplus$ is the image dilation operator.

Supplementary Figures

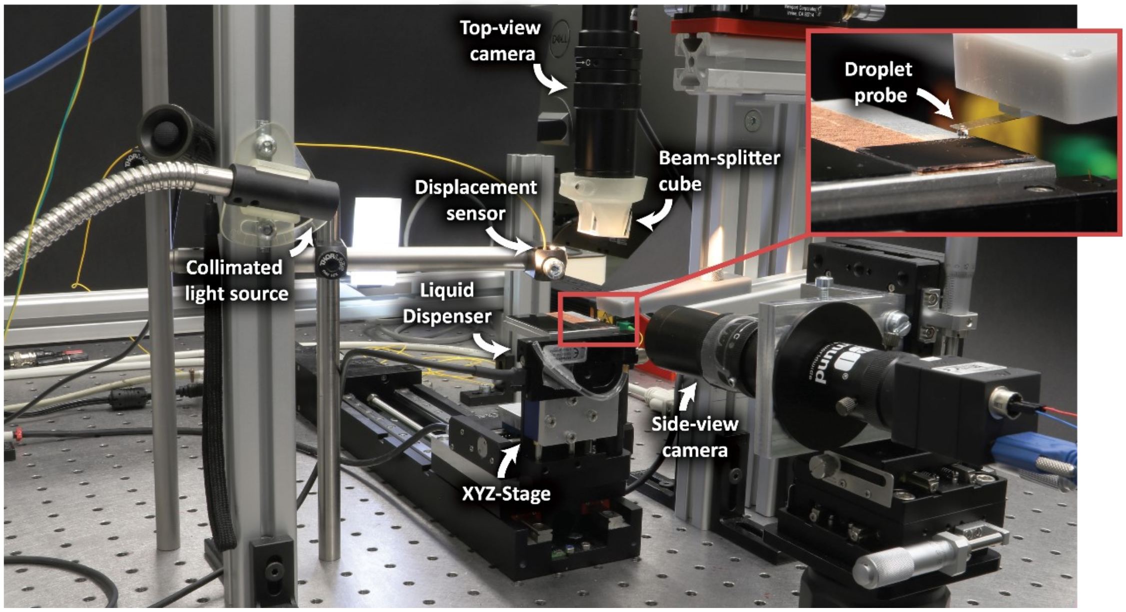

Fig. S2 Photograph of the experimental setup. The inset shows the droplet probe consisting of a water droplet hanging from the transparent holding disk.

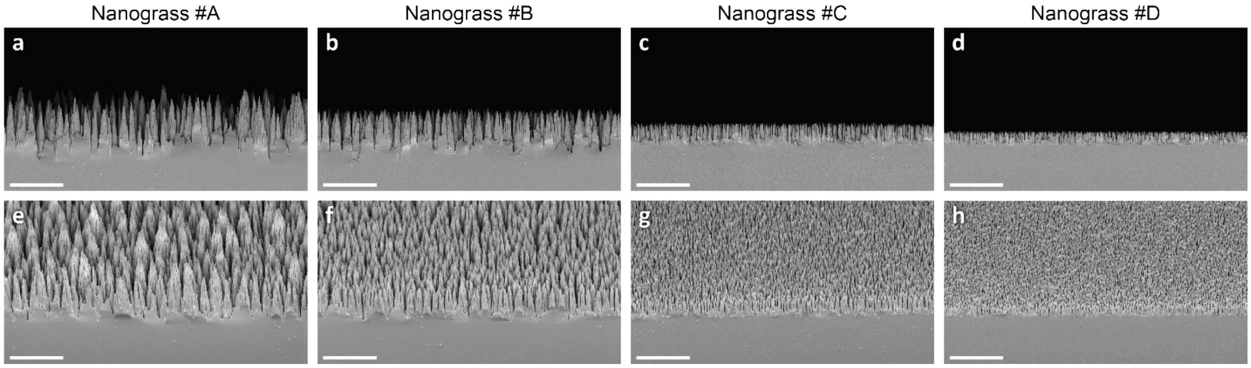

Fig. S3 SEM micrographs of the different types of nanograss. Scale bars 5 µm. (**a**-**d**) cross-section view. (**e**-**h**) perspective view (45 °).

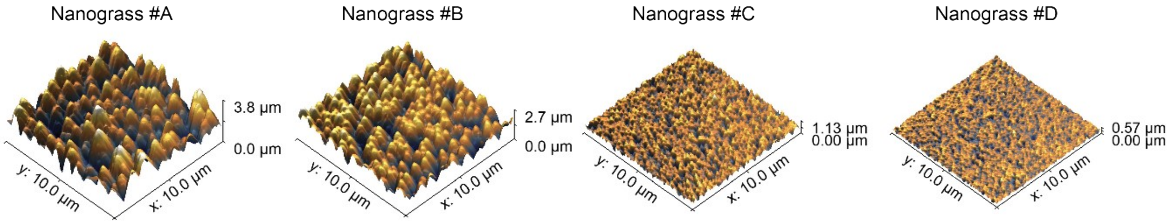

Fig. S4 AFM measurements of different types of nanograss. Z-axes to scale.

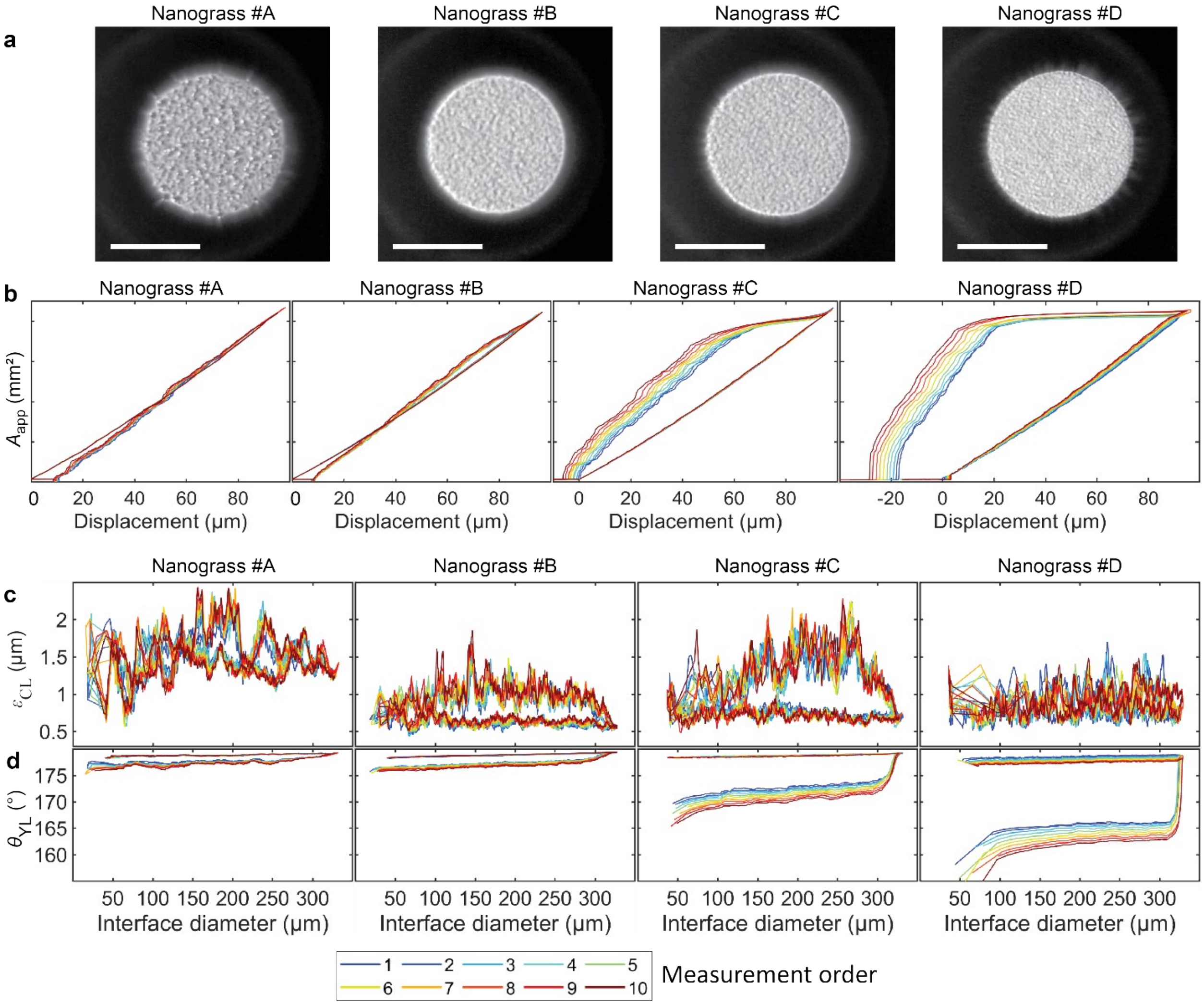

Fig. S5 Raw data of different types of nanograss for 10 measurements at the same location: (**a**) Top-view snapshots taken when the interface area was the largest, i.e. before the motion of sample stage reverses direction. (**b**) Apparent contact area, A_{app}, as a function of sample stage displacement. (**c**) Apparent contact line irregularity, ε_{CL}, and (**d**) modelled contact angle, θ_{YL}, as a function of interface diameter.

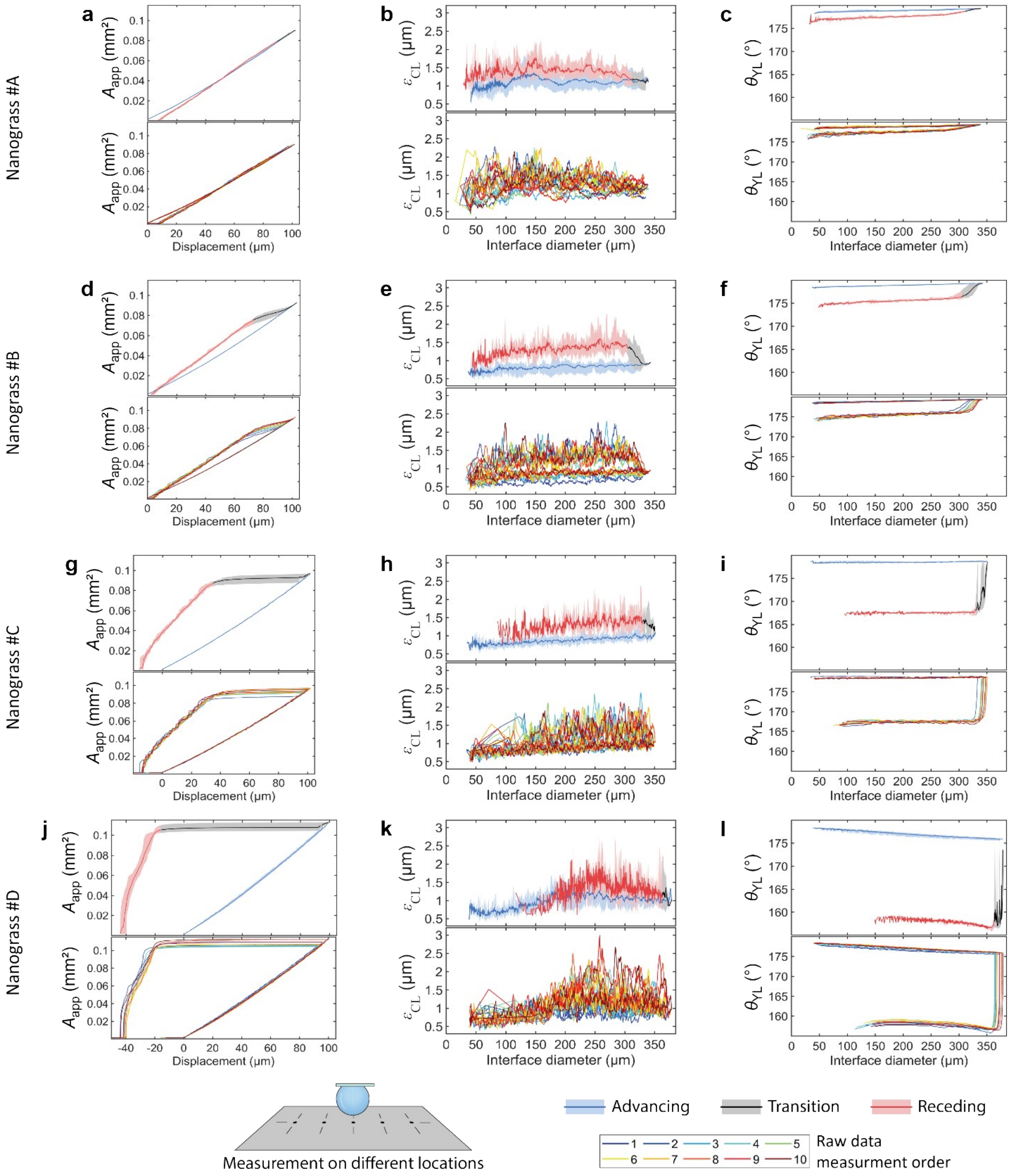

Fig. S6 Apparent contact area, A_{app}, contact line irregularity, ε_{CL}, and modelled contact angle θ_{YL} obtained at 10 different locations for each type of silicon nanograss samples. For each quantity, the top plot shows the mean value in solid line and shaded area represents minimum and maximum values observed. Bottom plots show raw data. (**a**) A_{app} as a function of sample stage displacement for nanograss #A. (**b**) ε_{CL} as a function of interface diameter. (**c**) θ_{YL} as a function of interface diameter. (**d-f**) A_{app} , ε_{CL}, and θ_{YL} for nanograss #B. (**g-i**) A_{app}, ε_{CL}, and θ_{YL} for nanograss #C. (**j-l**) A_{app}, ε_{CL}, and θ_{YL} for nanograss #D.

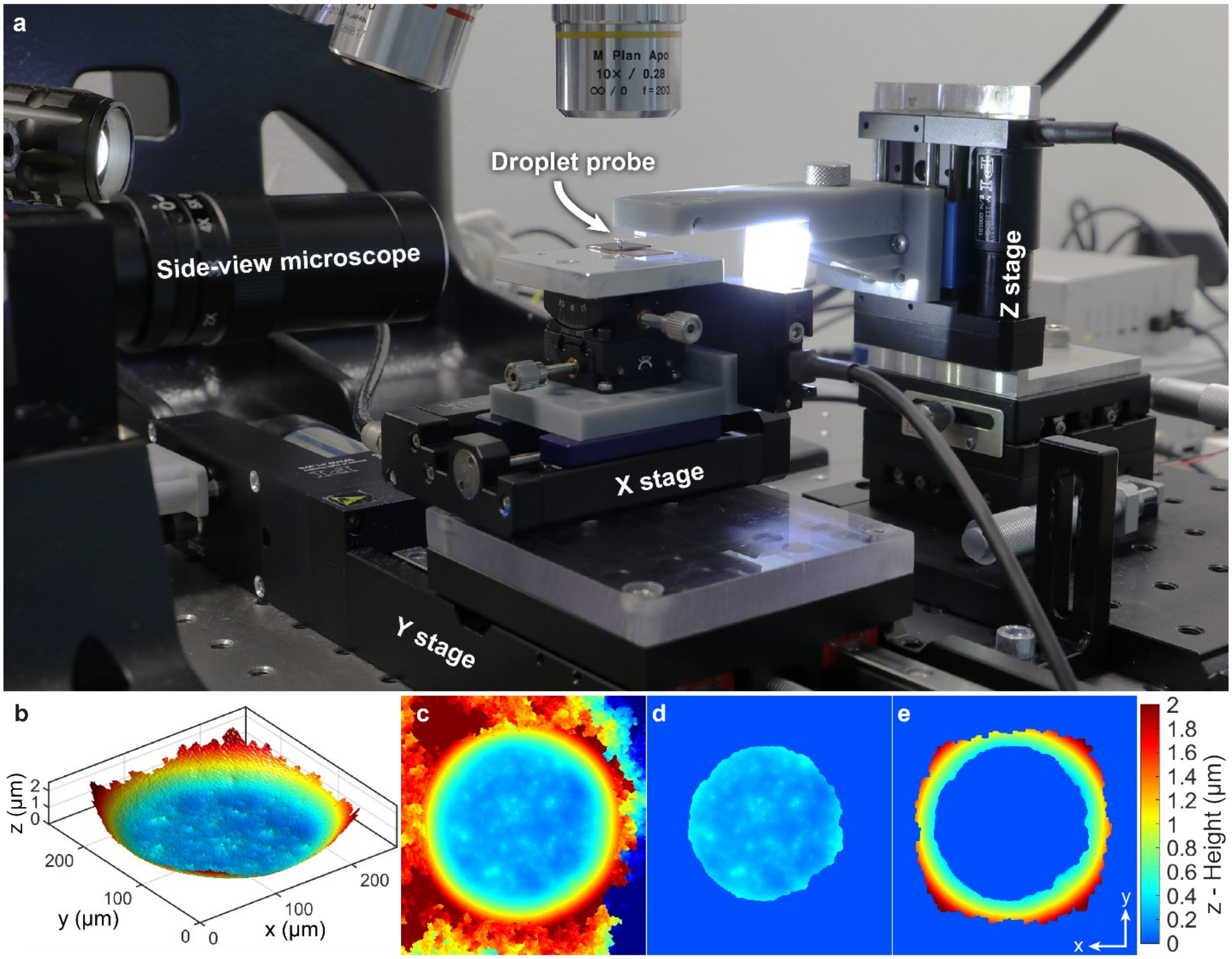

Fig. S7 Digital Holography Microscope experimental setup for verification of the contact angles on the different types of nanograss. (**a**) Experimental setup. A long working distance lens is used on the DHM, 10x, NA 0.28. An XY stage is used to move the sample stage (motors model M-404.4PD and M-122.2DD1, Physik Instrumente GmbH, Germany). The probe is mounted on a precision M-111.1DG for Z-axis displacement. A 2-DOF tilt stage is used to level the sample surface to XY motor motion plane. The droplet is formed with a PipeJet from BioFluidix GmbH, by dispensing purified water. (**b**) 3D representation of wetting interface. (Z-axis was scaled by 20x) (**c**) Example raw data of a single frame acquired on nanograss #A. The data is then processed to isolate the interface (**d**) and the meniscus (**e**). The data is levelled by a plane fitted to the interface. 300 radial slices are taken around the meniscus data, to each a quadratic fit is performed. The contact angle is measured as the angle of the quadratic fit at the intersection with the z=0 plane. The mean and standard deviation of the contact angles are presented in Table S2 as θ_{DHM} for each type of nanograss.

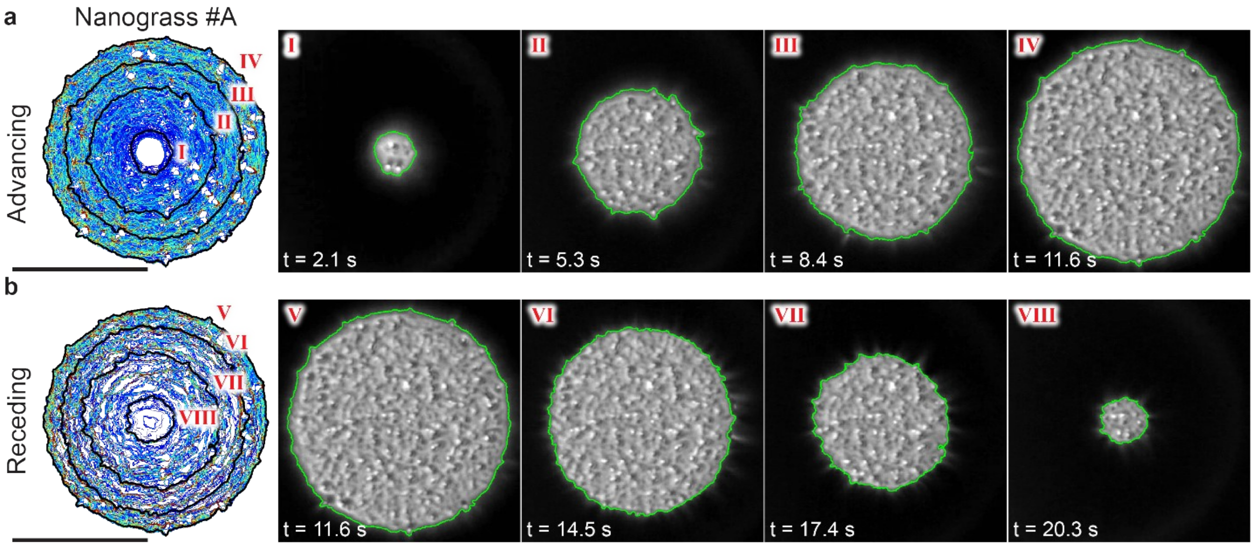

Fig. S8 Interface contact line snapshots at four different times during advancing and receding for one measurement in nanograss #A. Black lines mark position of CL at each time. (**a**) During advancing and (**b**) during receding. Scale bars: 200 μm

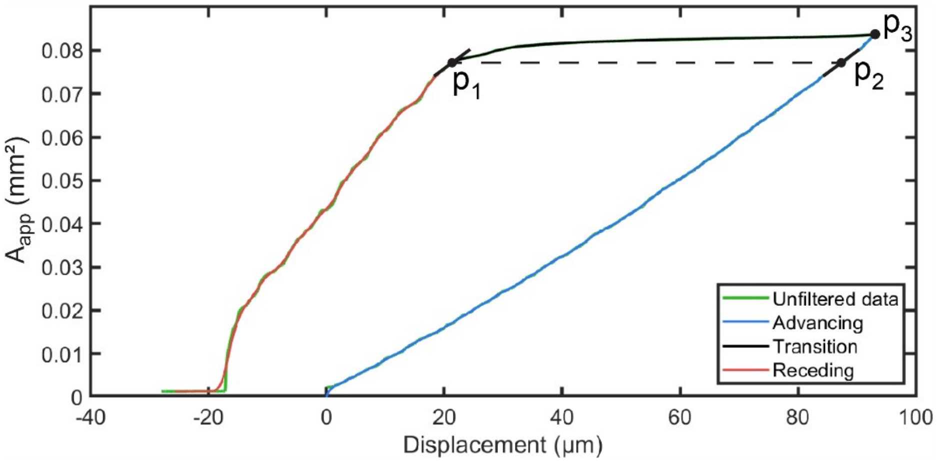

Fig. S9 Illustration of the calculation of the transition phase. The curve is an example of apparent contact area as a function of the displacement measurement for nanograss #D. The points defining the transition phase are marked. The point where the sample stage reverses direction is marked p_3. To determine the end of the transition phase, p_1, the data was first filtered. Then p_1 was found as the point where the rate of change in area during receding was 5% larger than the rate of change for the equivalent area during advancing, p_2.

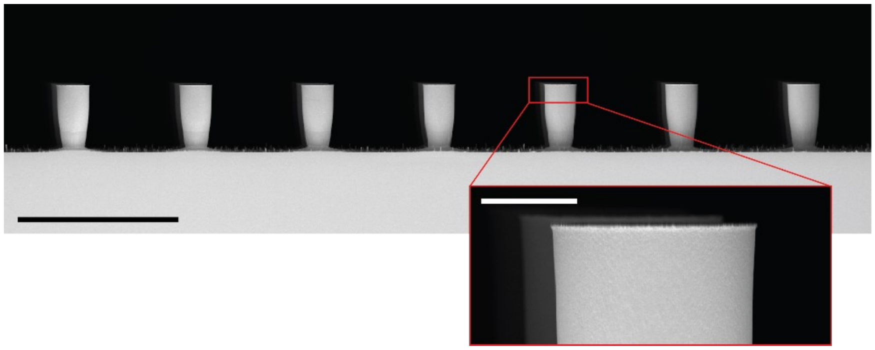

Fig. S10 Side-view of SEM micrograph of micropillars sample. Diameter: 20 μm; Period: 80 μm; Height: 44 μm. Main scale bar: 100 μm; Inset scale bar: 10 μm

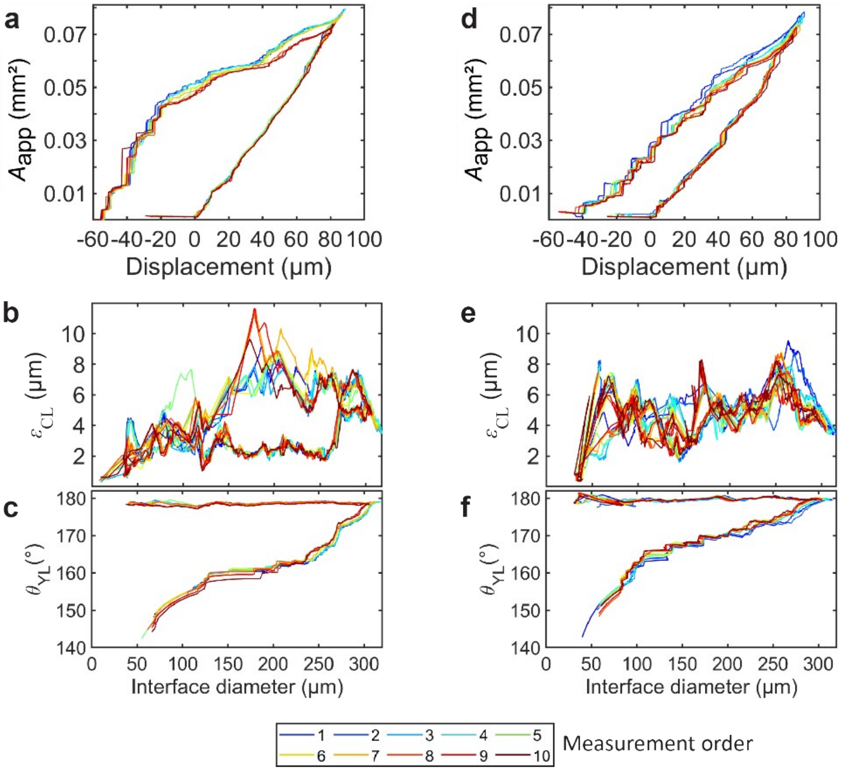

Fig. S11 Apparent contact area, A_{app}, contact line irregularity, ε_{CL} , and modelled contact angle θ_{YL} on plant leaves for 10 repetitions. (a) A_{app} for the Maranta leaf (b) ε_{CL}, for the Maranta leaf. (c) θ_{YL} for the Maranta leaf. (d) A_{app} for the Musa leaf (e) ε_{CL} for the Musa leaf. (f) θ_{YL} for the Musa leaf.

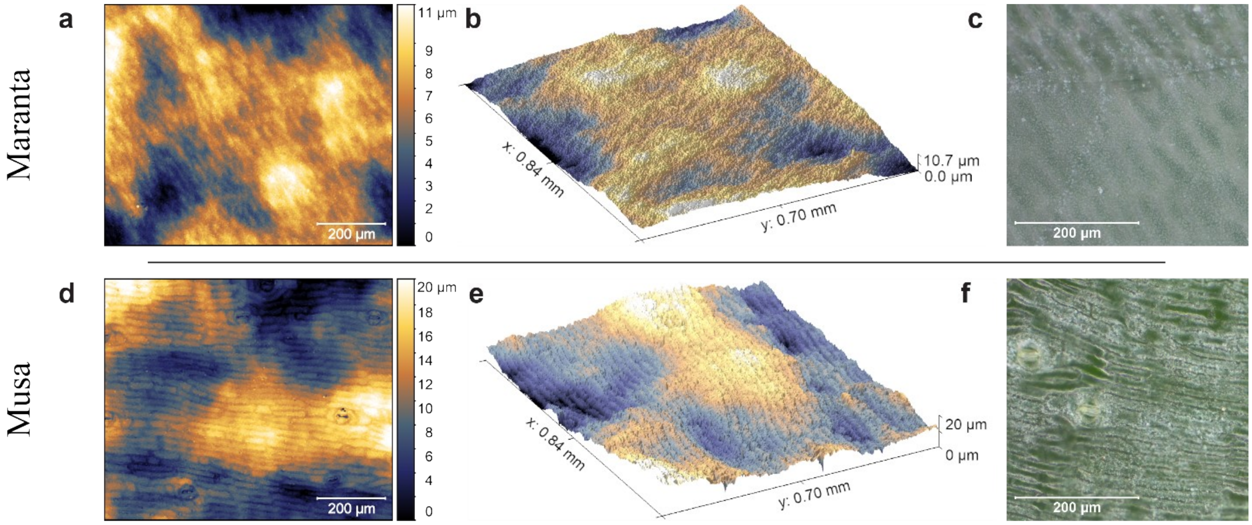

Fig. S12 Microscope images of the surfaces of the Maranta and Musa plant leaves. (a–b) Maranta leaf surface confocal image, (c) Maranta leaf optical micrograph. (d–e) Musa leaf surface confocal image, (f) Musa leaf optical micrograph.

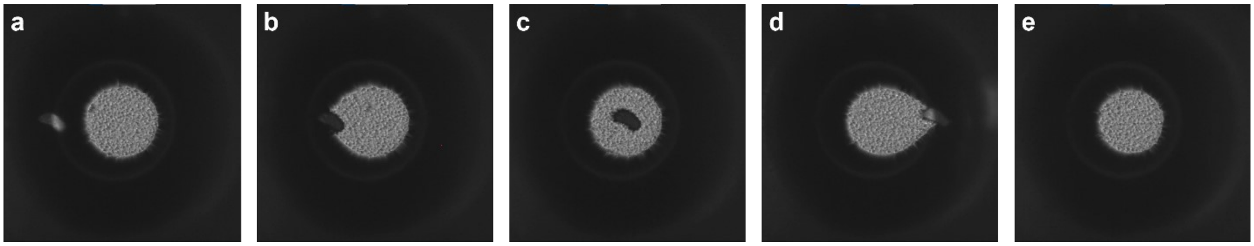

Fig. S13 Evolution of the apparent wetting interface when the droplet probe is sliding on a silicon nanograss sample with a dot-like scratch. (**a**) before touching, (**b**) when the left edge of the interface touches the scratch, (**c**) when the scratch is inside the interface; (**d**) when the scratch is leaving the interface; (**e**) when the scratch has left the interface.

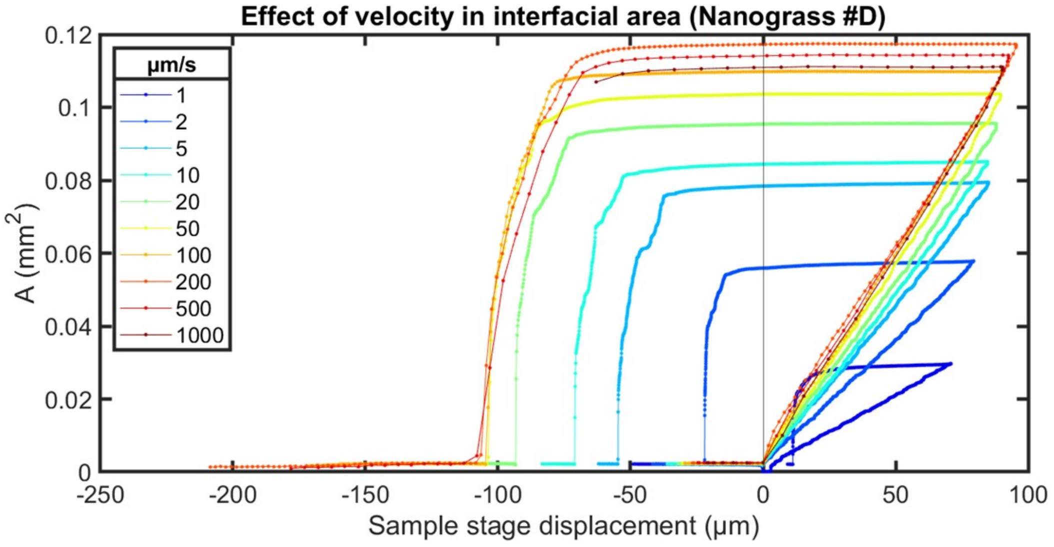

Fig. S14 Raw measurements on Nanograss #D with different sample stage velocities. At slower velocities, below 5 μm/s the effects of evaporation are significant and the experiment duration is also longer. At speeds above 20 μm/s the advancing and receding phases show less details of interaction with the surface. Also, at higher speeds the density of points is significantly reduced, acquired at 100 Hz.

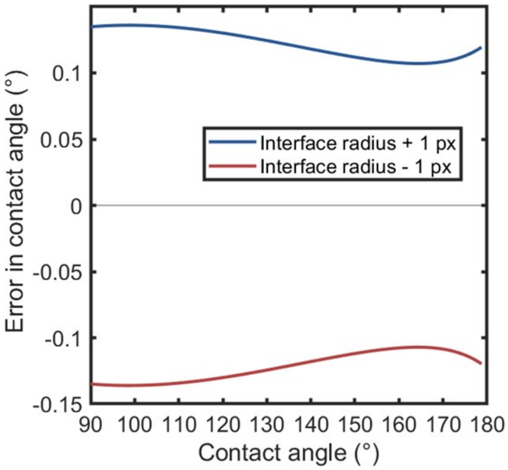

Fig. S15 Contact angle errors as a function of contact angle for one-pixel error in interface radius estimation. The interface radius is estimated from top-view image analysis and used in the Young-Laplace model to calculate θ_{YL}. The uncertainty remains bounded near the contact angle of 180°, while in conventional CAG the contact angle errors grow to several degrees near 180 ° (see Fig. 8 in reference 4 for the comparative plot).

Supporting Tables

Table S1 Surface properties of each different silicon nanograss type. Etch depth and spike tip radius were measured manually using side-view SEM measurements. Spike density was calculated from AFM images, with spike spacing being the square root of the inverse of spike densities.

	**Nanograss #A**	**Nanograss #B**	**Nanograss #C**	**Nanograss #D**
**Etch depth (µm)**	5.0 ± 0.6	3.5 ± 0.5	1.8 ± 0.2	1.1 ± 0.10
**Spike tip radius (nm)**	29 ± 8	34 ± 11	32 ± 6	40 ± 10
**Spike density (µm^{-2})**	1.0	1.4	14	18
**Spike spacing (µm)**	1.0	0.85	0.27	0.24

Table S2 Contact angle for each type of nanograss measured with different methods (mean ± standard deviation). θ_{YL} – Mean contact angle from Young-Laplace simulation for measurements in the same locations. θ_{DHM} – Contact angle measured with DHM. θ_{CAG} – Measured with commercial contact angle goniometer.

	**#A**		**#B**		**#C**		**#D**	
	Advancing	Receding	Advancing	Receding	Advancing	Receding	Advancing	Receding
**θ_{YL} (°)**	178.9 ± 0.2	177.7 ± 0.5	179.0 ± 0.2	177.3 ± 0.4	178.8 ± 0.2	171.9 ± 0.7	178.0 ± 0.3	164.3 ± 0.4
**θ_{DHM} (°)**	179.2 ± 0.3	178.8 ± 0.5	179.3 ± 0.3	178.6 ± 0.4	179.1 ± 0.2	-	178.3 ± 0.5	-
**θ_{CAG} (°)**	171 ± 4	170 ± 6	170 ± 1.7	171 ± 2	171 ± 1.7	172 ± 1.0	170 ± 2	167 ± 3

Table S3 Mean apparent contact line irregularity, ε_{CL}, during advancing and receding phases, on each type of nanograss. Values were measured 10 times on same location and different locations. ANOVA analysis F-value and p-value of the measurements are also shown.

		**Nanograss #A**	**Nanograss #B**	**Nanograss #C**	**Nanograss #D**	**F-value**	**p-value**
**Same location**	**Adv. ε_{CL} (µm)**	1.4 ± 0.17	0.62 ± 0.06	0.72 ± 0.07	0.8 ± 0.10	1460	1.2×10^{-37}
	**Rec. ε_{CL} (µm)**	1.5 ± 0.4	1.0 ± 0.17	1.3 ± 0.4	1.0 ± 0.15	225	2.2×10^{-23}
**10 diff. locations**	**Adv. ε_{CL} (µm)**	1.1 ± 0.17	0.83 ± 0.08	0.9 ± 0.11	1.0 ± 0.2	37	3.8×10^{-11}
	**Rec. ε_{CL} (µm)**	1.3 ± 0.3	1.2 ± 0.4	1.2 ± 0.4	1.1 ± 0.5	6	2.8×10^{-3}

Table S4 Advancing and receding contact angles for micropillar sample, performed with a commercial contact angle goniometer.

	**Micropillar surface**	
	Advancing	Receding
**θ_{CAG} (°)**	168 ± 3	154 ± 5

Table S5 Surface roughness of Maranta and Musa leaves. Sa – Mean surface roughness, calculated for the whole surface of each leaf in Fig. S8. Ra – Mean line roughness, measured from six lines drawn at random orientations.

	**Maranta**	**Musa**
**Mean surface roughness – S_a (µm)**	1.7	3.4
**Mean line roughness - R_a (µm)**	1.4	3.3

Table S6 Advancing and receding wetting properties for Maranta and Musa leaves for 10 repetitions, measured at the same location (mean ± standard deviation). ε_{CL} – mean apparent contact line irregularity. θ_{YL} – contact angle from Young-Laplace simulation. θ_{CAG} – mean advancing and receding contact angle measurements, obtained with a commercial contact angle goniometer.

	**Maranta**		**Musa**	
	Advancing	Receding	Advancing	Receding
**ε_{CL} (µm)**	3 ± 1.1	6 ± 2	5 ± 1.3	5 ± 1.1
**θ_{YL} (°)**	178.6 ± 0.3	159 ± 4	179.7 ± 0.4	168 ± 5
**θ_{CAG} (°)**	166.9 ± 3.7	166.4 ± 4.2	166.5 ± 4.0	167.7 ± 1.7

Supporting Videos

Movie S1 (separate file) Contact area, contact line irregularity and contact angle on four different silicon nanograss substrates. The measurements were carried out for silicon nanograss #A, #B, #C, and #D. The green line shows the detected interface outline. Area and apparent contact line irregularity plots are shown in real-time. Video contrast and brightness were adjusted.

Movie S2 (separate file) Pinning and depinning on silicon pillars with different droplet-sample alignments. Three cases were measured: centered on one pillar, between four pillars, and between two pillars. Video contrast and brightness were adjusted.

Movie S3 (separate file) Contact area and contact line irregularity on Maranta and Musa plant leaves. The green line shows the interface outline. Area and apparent contact line irregularity plots are shown in real-time. Video contrast and brightness were adjusted.

Movie S4 (separate file) Lateral scan measurement on scratched silicon nanograss #A samples. Two cases were measured, a dot-like scratch and a line-like scratch. Video contrast and brightness were adjusted.

References

1 David Whitehouse, in *Surfaces and their Measurement*, Hermes Penton Science, 2002, pp. 48–95.

2 N. Otsu, *IEEE Trans Syst Man Cybern*, 1979, **9**, 62–66.

3 M. Kass, A. Witkin and D. Terzopoulos, *Int J Comput Vis*, 1988, **1**, 321–331.

4 M. Vuckovac, M. Latikka, K. Liu, T. Huhtamäki and R. H. A. Ras, *Soft Matter*, 2019, **15**, 7089–7096.
